# Intravascular Squamous Cell Carcinoma Metastases Presenting Solely as Retiform Purpura: A Case Report

**DOI:** 10.7759/cureus.106290

**Published:** 2026-04-01

**Authors:** Eleni Pilitsi, Anne M Stowman, Charles Ashley, Melanie R Bui

**Affiliations:** 1 Department of Dermatology, University of Vermont Medical Center, South Burlington, USA; 2 Department of Pathology and Laboratory Medicine, University of Vermont Medical Center, South Burlington, USA; 3 Division of Gynecologic Oncology, University of Vermont Medical Center, South Burlington, USA

**Keywords:** cancer immunotherapy, cutaneous metastases, metastatic cancer, retiform purpura, squamous cell cervical carcinoma

## Abstract

Herein, we report the case of a woman in her mid-forties with stage IIB cervical squamous cancer status post-chemoradiation and brachytherapy with curative intent one year prior, who subsequently presented with lacy red to violaceous patches on the forehead, abdomen, back, and legs, as well as mildly tender, ill-defined, purpuric dermal nodules on the right upper back and right inner thigh. Hypercoagulability work-up was negative, and biopsy was consistent with metastatic, intravascular squamous cell carcinoma of cervical origin. This prompted repeat imaging, which was suspicious for metastatic disease to lymph nodes, lungs, and pericardium with multiple bilateral acute/subacute embolic brain infarcts and left ventricular thrombus, and the patient was subsequently started on pembrolizumab, carboplatin, and paclitaxel. Dermatology recommended warming the skin during infusions to promote vasodilation and improve intravascular medication distribution. Despite almost complete resolution of purpuric skin findings and initial improvement in metastatic bone and lymph node disease, the patient had disease progression with worsening bone and new leptomeningeal metastases, as well as subcutaneous metastatic nodules, and was switched to second-line immunotherapy, tisotumab and bevacizumab. Unfortunately, the patient continued to have progressive metastatic disease, leading to multiple complications and hospitalizations, and was ultimately transitioned to hospice care. She passed eleven months post-presentation. Our case highlights the importance of considering retiform purpura as a rare manifestation of intravascular metastases in patients with cervical squamous cell carcinoma, the value of cutaneous metastases in the management of oncologic patients, and the need to further assess the prognostic utility of cutaneous metastases in the era of immunotherapy, which, based on our case, appears to remain a poor prognostic sign.

## Introduction

Cutaneous metastases secondary to cervical squamous cell carcinoma are rare, and their incidence increases as tumor stage advances (0.8-4.8%) [1, 2]. The most common morphologic patterns of metastatic cervical squamous cell carcinoma are nodules and plaques, followed by inflammatory telangiectatic macules and papular eruption [3]. The most common sites of cutaneous metastases are the abdominal wall and vulva, followed by the anterior chest wall, but any site may be involved [4, 5]. Direct and lymphatic spread are the most likely modes of metastasis, though there is a single report of presumed hematogenous spread implicated in cutaneous hand nodules metastatic from cervical carcinoma [6]. Herein, we present a case of intravascular cervical squamous cell carcinoma metastatic to the skin presenting solely as retiform purpura. This article was previously presented as an oral abstract at the 2025 AAD Annual Meeting Gross & Microscopic Symposium on March 8, 2025. Retiform purpura is a clinical sign resulting from cutaneous blood vessel ischemia and is comprised of non-blanching, purpuric patches or plaques that form angulated or branching patterns. The differential diagnosis of retiform purpura is broad and includes damage to the vessel wall (vasculitides of the small and medium vessels, depositional diseases, and angioinvasive infections) or lumen occlusion (hypercoagulable states, intravascular malignancy, and embolic disease).

## Case presentation

**Figure 1:**
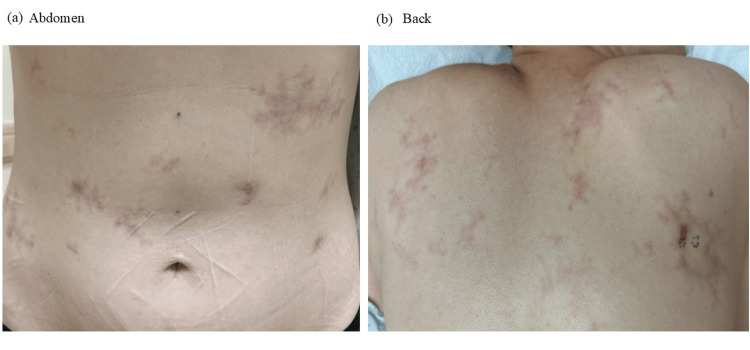
Clinical presentation of metastatic intravascular squamous cell carcinoma. (a) Scattered lacy violaceous patches on the abdomen. (b) Scattered lacy violaceous patches on the back with a purpuric nodule on the right midback, which was biopsied.

A woman in her mid-forties was referred to the dermatology clinic for a lacy red rash of two months' duration. The rash started on the abdomen and, over the course of two months, spread to the back, forehead, and thighs. It was associated focally with pain. Review of systems was positive for migraine headaches and fatigue of three weeks' duration. The patient had a history of stage IIB, programmed death-ligand 1-positive, cervical squamous cell carcinoma status post-chemoradiation and brachytherapy with curative intent a year prior to presentation. She underwent a modified radical hysterectomy, bilateral salpingo-oophorectomy, and paraaortic lymph node dissection for residual disease, followed by adjuvant chemoradiation to her para-aortic lymph nodes five months prior to presentation. At that time, the patient declined adjuvant chemotherapy and immunotherapy and had evidence of nodal disease resolution three months post-chemoradiation. On exam, she was thought to be in remission, but there were lacy red-to-violaceous patches on the forehead, abdomen (Figure 1a), back, and legs. There were ill-defined, purpuric, dermal nodules with mild tenderness to palpation (Figure 1b). A complete blood count and liver and kidney function panels were normal.

**Figure 2:**
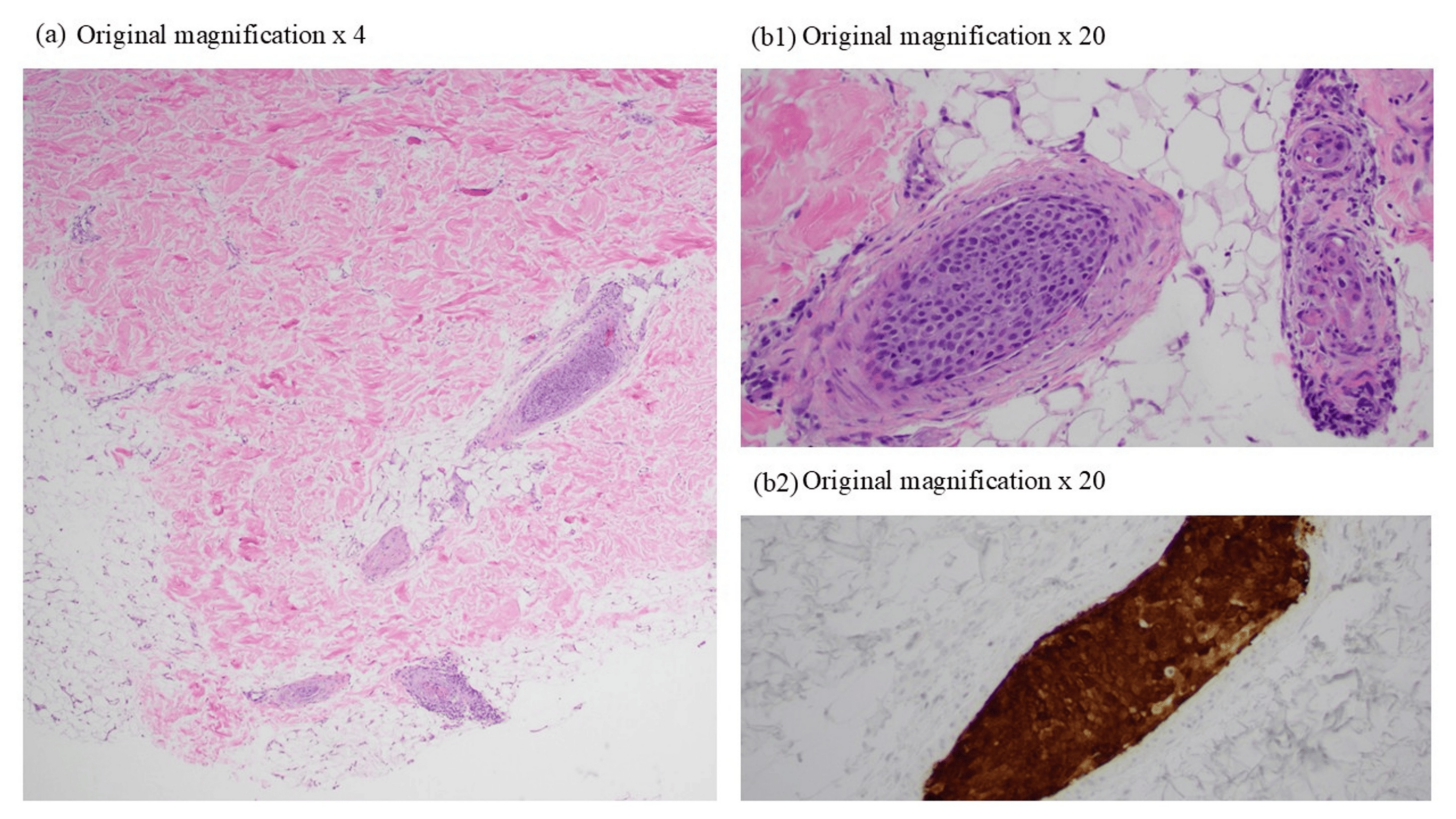
Histopathologic features of metastatic intravascular squamous cell carcinoma. (a) 4 mm punch biopsy from the right midback demonstrates multiple foci of intravascular collections of atypical squamous cells in the deep dermis and subcutaneous tissue (hematoxylin and eosin stain, 4x). (b1) High-power image of the intravascular collections of atypical squamous cells filling and expanding multiple vessels in the deep dermis (H&E, 20x) and superficial subcutis. (b2) P16 immunohistochemical stain strongly highlights the intravascular squamous cells (20x).

A punch biopsy from the right midback revealed intravascular collections of atypical squamous cells (Figure 2a and 2b1), keratin, and p16-positive (Figure 2b2) with similar morphology to the prior cervical biopsy, consistent with metastatic, intravascular squamous cell carcinoma of cervical origin.

**Figure 3:**
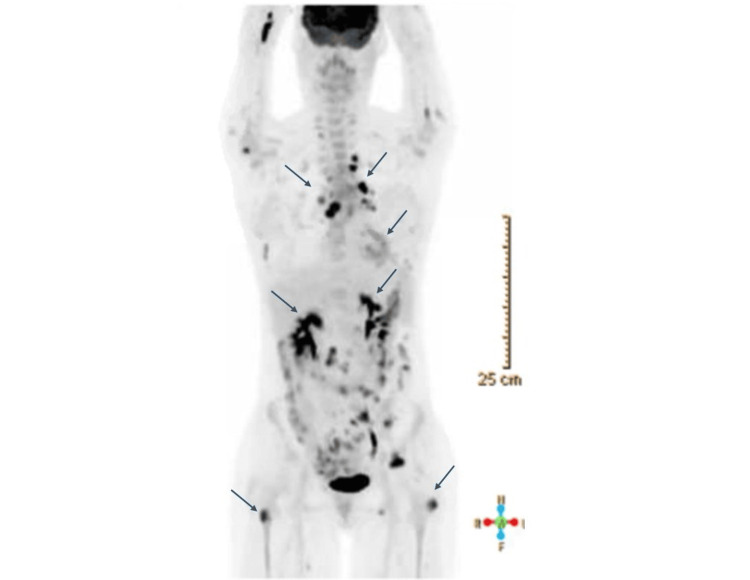
PET/CT scan demonstrates widely metastatic disease. Arrows depict metabolically active lymphadenopathy involving mediastinal, hilar, and mesenteric lymph nodes; multiple osseous metastases, including bilateral femur; and peripheral consolidative opacities in the left lower lobe with associated increased radiotracer uptake consistent with metastatic disease.

Subsequently, the patient was found to have multiple bilateral acute/subacute embolic brain infarcts. Hypercoagulability work-up was unremarkable. Lumbar puncture and cerebrospinal fluid analysis did not reveal malignant cells; therefore, the infarcts were attributed to underlying malignancy-associated hypercoagulability. The patient also developed a large malignant pericardial effusion with left ventricular thrombus. Repeat PET/CT (Figure 3) revealed extensive metastatic disease.

The patient was started on first-line chemo- and immunotherapy with carboplatin, paclitaxel, and pembrolizumab, and despite some initial treatment response after three cycles, her disease progressed after six cycles. Interestingly, while the patient’s retiform purpura markedly improved, she developed two discrete 0.8-1.5 cm skin-colored subcutaneous nodules on the scalp and one ~0.8 cm on the right back, which were biopsy-proven metastatic cervical squamous cell carcinomas. The patient was switched to tisotumab and bevacizumab, but unfortunately, her metastatic disease continued to progress. With many complications and several admissions thereafter, she was transitioned to hospice and passed eleven months after her initial presentation.

## Discussion

Herein, we discuss a unique case of retiform purpura as the sole presenting symptom of metastatic cervical squamous cell carcinoma. Our patient, who was thought to be in remission, developed a combination of clinical features suggestive of metastases, with the leading indicator of intravascular metastases being in the skin in the form of retiform purpura. She later manifested a malignant pericardial effusion, intravascular thrombosis of the left ventricle, and brain emboli as well as subcutaneous, osseous, lymphatic, visceral, and leptomeningeal metastases.

Cervical cancer most commonly metastasizes to the lungs, bones, liver, brain, ovaries, and lymph nodes, but only rarely to the skin (<2%) [7]. Stage IIIb has been reported as the most common clinical stage to present with cutaneous metastases [8]. The majority of metastasized cervical carcinomas to skin were histopathologically confirmed squamous cell carcinomas (63.8%), followed by adenocarcinomas (10.6%) [8].

Thrombotic and coagulopathic disorders, vasculitides, calciphylaxis, and angioinvasive infections can manifest with the morphology of retiform purpura [9]. Skin biopsy is essential to narrow down the etiology for this embolic phenomenon, as well as to characterize any suspicious lesion or rash in a patient with an underlying malignancy [5].

The mean interval between the diagnosis of the primary tumor and skin metastases is 20.7 months (range 0-120) [8]. Cutaneous metastasis has traditionally been considered a poor prognostic sign with a survival of 6-12 months, as it is often accompanied by metastases to other organs such as the brain, lung, and bone, like in our case [2]. Management tends to be palliative and may include radiation, chemotherapy, or surgery, either alone or in combination [8]. More recently, molecular testing and targeted immunotherapy have become widely available for the management of metastatic cervical cancer, and therefore, the prognosis of cutaneous metastases may change, especially in patients without otherwise extensive metastatic disease [10, 11]. In our case, chemotherapy and immunotherapy only temporarily halted metastatic disease progression. Additionally, regional hyperthermia has been reported to boost cytotoxic effects when combined with chemotherapy, and electrochemotherapy, a tool that combines chemotherapy with electric pulses, has been used to safely treat cutaneous metastases of various origins [12, 13]. However, these modalities are not widely available; thus, our patient was advised to warm her skin during chemotherapy to potentially increase diffusion to distant cutaneous sites.

## Conclusions

This is a rare and unique case of retiform purpura as the sole presenting symptom of metastatic cervical squamous cell carcinoma, which was diagnosed by skin biopsy alone. This case highlights the importance of skin examination in patients with a history of malignancy in remission; the need to consider cutaneous intravascular metastases within the differential for retiform purpura, even in otherwise well-appearing individuals; and the potentially poor prognosis related to cutaneous metastases even in the era of immunotherapy.

## References

[1] Benoulaid M, Elkacemi H, Bourhafour I (2016). Skin metastases of cervical cancer: two case reports and review of the literature. J Med Case Rep.

[2] Imachi M, Tsukamoto N, Kinoshita S, Nakano H (1993). Skin metastasis from carcinoma of the uterine cervix. Gynecol Oncol.

[3] Freeman CR, Rozenfeld M, Schopflocher P (1982). Cutaneous metastases from carcinoma of the cervix. Arch Dermatol.

[4] Hayes AG, Berry AD 3rd (1992). Cutaneous metastasis from squamous cell carcinoma of the cervix. J Am Acad Dermatol.

[5] Alrefaie SI, Alshamrani HM, Abduljabbar MH, Hariri JO (2019). Skin metastasis from squamous cell carcinoma of the cervix to the lower extremities: case report and review of the literature. J Family Med Prim Care.

[6] Pertzborn S, Buekers TE, Sood AK (2000). Hematogenous skin metastases from cervical cancer at primary presentation. Gynecol Oncol.

[7] Guo L, Liu Y, Zhang S, Liu W (2024). Case report: Cutaneous metastasis of squamous cervical carcinoma: complete regression after molecular diagnosis. Front Immunol.

[8] Agrawal A, Yau A, Magliocco A (2010). Cutaneous metastatic disease in cervical cancer: a case report. J Obstet Gynaecol Can.

[9] Georgesen C, Fox LP, Harp J (2020). Retiform purpura: a diagnostic approach. J Am Acad Dermatol.

[10] Mahapatra BR, Muraleedharan A, Badajena A, Das Majumdar SK, Haroon KMN (2023). Cutaneous metastasis in a treated case of cervical cancer With extraordinary response to chemotherapy: a case report of a rare event and review of the literature. Cureus.

[11] Shen Y, Jin X, Zhu Y, He C (2024). Clinical analysis of patients with skin metastasis of cervical squamous cell carcinoma. Chin Clin Oncol.

[12] Richtig E, Hoff M, Rehak P (2003). Efficacy of superficial and deep regional hyperthermia combined with systemic chemotherapy and radiotherapy in metastatic melanoma. J Dtsch Dermatol Ges.

[13] Claussen CS, Moir G, Bechara FG (2022). Prospective cohort study by InspECT on safety and efficacy of electrochemotherapy for cutaneous tumors and metastases depending on ulceration. J Dtsch Dermatol Ges.

